# Editorial: Low carbon economy and health in the context of carbon neutrality

**DOI:** 10.3389/fpubh.2023.1273204

**Published:** 2023-09-12

**Authors:** Yuanjun Zhao, Zheng Liu, Chunjia Han

**Affiliations:** ^1^School of Accounting, Nanjing Audit University, Nanjing, China; ^2^School of Management, Shanghai University of Engineering Science, Shanghai, China; ^3^Department of Management Birkbeck, University of London, London, United Kingdom

**Keywords:** public health, low-carbon economy, carbon neutrality, climate change, environmental protection

With climate change becoming a global concern, a low-carbon economy has become an inevitable trend. Carbon neutrality is considered an important step in curbing global greenhouse gas emissions and reducing carbon footprints. However, a low-carbon economy is not only about environmental protection but also closely related to human health. Against this background, a special topic on low-carbon economy and health in the context of carbon neutrality is proposed, aiming to promote the emergence of a series of emerging research and the sustainable development of low-carbon economy and human health. The articles included under this topic have been researched from different perspectives, introducing new research perspectives, methods, and techniques for the further development of the topic, which will have a positive impact on promoting the in-depth research and development of the topic.

The topic of low-carbon economy and health in the context of carbon neutrality has been studied multidimensionally in the articles included in this topic. In terms of the doctor-patient relationship, Zhu et al. concluded that the NSGA-II algorithm performs well in the doctor-patient bilateral matching problem, but the matching accuracy needs to be further improved. In terms of the pharmaceutical supply chain, Fu and Zhao considered the risk of pharmaceutical supply chain disruption under two modes of centralized and decentralized decision-making and constructed a combined contract model to effectively coordinate the pharmaceutical supply chain under supply disruption crises, but further research is needed in the area of stable coordination of pharmaceutical supply chain with multi-levels and multi-objects. In terms of public health expenditures, Omri et al. concluded from their study that public and private health expenditure is effective in mitigating environmental degradation on health status in Saudi Arabia, especially public health expenditure. In terms of regional green development, Wang W et al. constructed a rating model for the high-quality development of China's Yangtze River Delta Green Integration Demonstration Zone, and proposed methods and approaches to measure the construction milestones of high-quality development, which provides a reference for the evaluation study of high-quality development in other city clusters. The model suitability can be further improved by combining the actual data of city clusters in future studies. In terms of government guidance, Xu et al. observed in their study that the characteristics of local government information sources (credibility, professionalism, and attractiveness) have a significant positive effect on consumers' willingness to purchase low-carbon agricultural products, which can effectively promote the development of low-carbon agriculture. However, the study lacks quantitative research on the degree of contribution of regional brand low-carbon agricultural products to the development of low-carbon agriculture. Hu et al. offer suggestions for establishing and managing a low-carbon technology innovation system, along with insights into theoretical research on public health and high-quality development in China. Wang Q et al. examine the impact mechanism of environmental education on environmental quality in the context of low-carbon economy. Wu et al. analyze the optimal use of urban resources based on public health. Bai et al. utilize unit root tests, co-integration tests, and regression analysis to empirically investigate the associations between carbon emissions and GDP in the industry, construction, and transportation sectors. Zheng et al. provide significant practical contributions to sustainable development and the promotion of responsible population growth.

To sum up, for the special topic of low-carbon economy and health in the context of carbon neutrality, researchers have carried out relevant research in the areas of healthcare resource allocation, public health resources, urban green and low-carbon development, and the process of low carbonization of sub-fields. The further deepening of this topic expands the research field, provides subdivided research directions, relevant theoretical foundations, research methodological references, data support, and case analyses, and plays a positive role in promoting subsequent in-depth research. In more in-depth research in the future, the following can be further explored: the evaluation system of healthcare resource allocation and the construction of a low-carbon supply chain, the supply and allocation of public health resources in less developed regions, the quantitative assessment method of the impact of low-carbon economic policies and measures on health, and the process of decarbonization in the fields of agriculture, industry, and urban construction, as well as synergistic effects among different fields.

## Author contributions

YZ: Writing—original draft. ZL: Writing—review and editing. CH: Writing—review and editing.

